# Combined Influence of Eight Lifestyle Factors on Metabolic Syndrome Incidence: A Prospective Cohort Study from the MECH-HK Study

**DOI:** 10.3390/nu16040547

**Published:** 2024-02-16

**Authors:** Yun-Yang Deng, Fei-Wan Ngai, Jing Qin, Lin Yang, Ka-Po Wong, Harry-Haoxiang Wang, Yao-Jie Xie

**Affiliations:** 1 School of Nursing, The Hong Kong Polytechnic University, Hong Kong SAR, China; yunyang.deng@polyu.edu.hk (Y.-Y.D.); vivian.ngai@polyu.edu.hk (F.-W.N.); harry.qin@polyu.edu.hk (J.Q.); l.yang@polyu.edu.hk (L.Y.); 2 Department of Applied Social Sciences, The Hong Kong Polytechnic University, Hong Kong SAR, China; portia.wong@polyu.edu.hk; 3 School of Public Health, Sun Yat-Sen University, Guangzhou 510080, China; wanghx27@mail.sysu.edu.cn; 4 College of Medicine and Veterinary Medicine, The University of Edinburgh, Edinburgh EH8 9AG, UK; 5 Research Centre for Chinese Medicine Innovation, The Hong Kong Polytechnic University, Hong Kong SAR, China

**Keywords:** lifestyle, lifestyle score, lifestyle index, combined lifestyle, metabolic syndrome

## Abstract

Although previous studies have shown significant associations between individual lifestyles and metabolic syndrome, limited studies have explored the combined effect of lifestyles. The purpose of this study was to investigate whether a combined lifestyle score was associated with metabolic syndrome incidence in Hong Kong Chinese women. This prospective cohort study included 1634 women (55.9 ± 8.6 years) without baseline metabolic syndrome, diabetes, myocardial infarction, or stroke. Eight lifestyle factors (smoking, physical activity, sedentary time, sleep, stress, fatigue, diet, and alcohol) were included by assigning 0 (unhealthy) or 1 point (healthy). The overall score was the sum of these points, ranging from 0 (the least healthy) to 8 points (the healthiest). Metabolic syndrome was diagnosed by the joint interim statement. During a 1.16-year follow-up, 179 (11.0%) new metabolic syndrome cases were identified. The incidences for the 0–3-point, 4-point, 5-point, and 6–8-point groups were 12.8% (79/618), 11.5% (42/366), 9.4% (29/309), and 8.5% (29/341), respectively. Compared to the lowest combined lifestyle score group, the highest group had a 47% reduced metabolic syndrome incidence, with an adjusted odds ratio and 95% confidence interval of 0.53 (0.33–0.86) (*p* = 0.010). These findings indicate that a higher combined lifestyle score was associated with a lower metabolic syndrome incidence in this population.

## 1. Introduction

Non-communicable diseases (NCDs) are the main contributor to morbidity and mortality [1]. Among the many NCDs, metabolic syndrome (MetS) has become an important public health concern, correlating with an increased risk of other major NCDs such as cardiovascular diseases (CVDs) and type 2 diabetes (T2D) [2]. MetS was characterized by abdominal adiposity, elevated levels of blood glucose, blood triglycerides, and blood pressure, along with reduced levels of high-density lipoprotein (HDL) cholesterol [1]. The global prevalence of MetS was estimated to be 25% in 2018 [1]. Furthermore, data reported a MetS prevalence of 34.7% among individuals aged ≥20 years in the United States between 2011 and 2016 [3]. Likewise, a prior study showed a standardized prevalence of 31.1% for MetS among Chinese residents aged ≥20 years during the period from 2015 to 2017 [4].

Previous investigations have revealed significant associations between individual lifestyles and MetS. For example, both active smoking and secondhand smoke exposure were positively associated with MetS [5,6]. A meta-analysis demonstrated that an increase of 10 metabolic equivalent of task (MET) h/week in leisure-time physical activity correlated with an 8% reduction in MetS incidence [7]. The relationship between sleep duration and MetS followed a J-shaped pattern, as revealed by a meta-analysis [8]. Moreover, numerous studies reported significant relationships between MetS and dietary habits, including both inverse associations (e.g., fruits and vegetables [9], soy protein [10], dairy products [11], fish [12], eggs [13], and pickled vegetables [14]) and positive correlations (e.g., red meats [12], processed meats [15], and cake [16,17]). Other MetS-related lifestyles encompassed alcohol [18], stress [19], and fatigue [20].

Nevertheless, recently, many investigations have been conducted to explore the correlations of combined lifestyle factors (usually measured as lifestyle scores/indices) with various NCDs including MetS [21,22,23,24,25,26,27,28,29]. For instance, a Spanish cohort study devised a nine-item lifestyle score and revealed an inverse association with MetS [28]. Similarly, an Iranian cohort study demonstrated an inverse relationship between a four-item lifestyle index (smoking, physical activity, obesity, and diet) and MetS [29]. These lifestyle scores/indices considered the potential interactions among lifestyles, providing a comprehensive foundation for policymakers to formulate health policies [21]. However, since lifestyles and disease characteristics are diverse across different ethnicities, these findings from other populations might not be applicable to Chinese people directly [21,22,23]. To the best of our knowledge, no cohort study has been undertaken to examine whether combined lifestyle factors are related to MetS in Chinese populations.

Therefore, this prospective cohort study aimed to investigate the association between an eight-item combined lifestyle score (CLS) and MetS incidence among Hong Kong Chinese women.

## 2. Materials and Methods

### 2.1. Study Population

**Figure 1:**
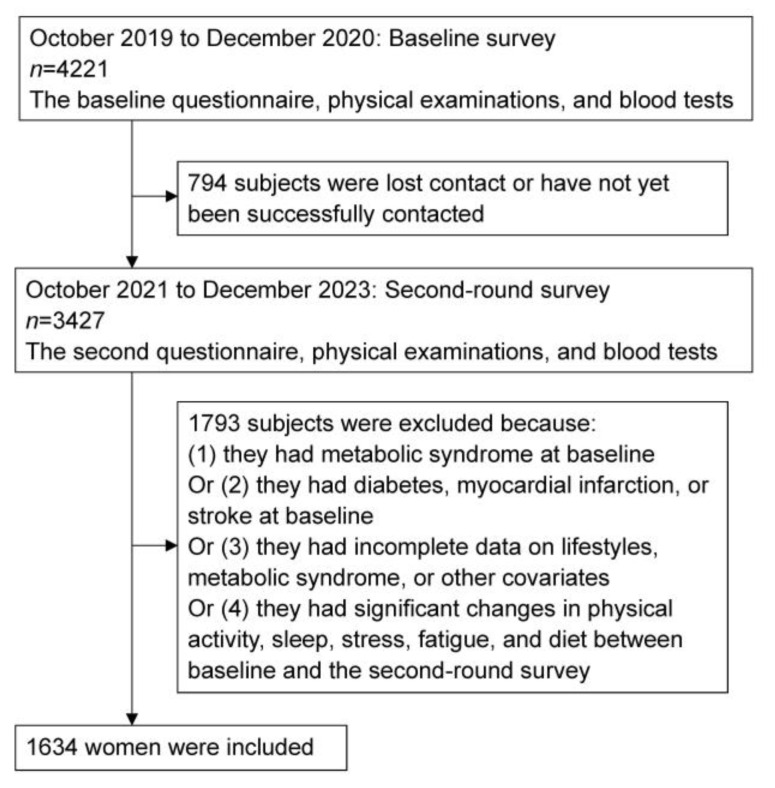
The flow chart of the present study.

This prospective cohort study was conducted based on the Migraine Exposures and Cardiovascular Health in Hong Kong Chinese Women (MECH-HK) cohort, which enrolled 4221 women aged ≥30 years at baseline (October 2019–December 2020) and followed up with 3427 women during the second-round survey (October 2021–December 2023). In this study, 1793 women who met one of the following criteria were excluded: (1) had MetS at baseline; (2) had MetS-related diseases at baseline, including diabetes, myocardial infarction, or stroke [2]; (3) had incomplete data on lifestyles, MetS, or other covariates; and (4) had significant changes (>±3 standard deviation (SD)) in physical activity, sleep, stress, fatigue, and diet between baseline and the second-round survey (Figure 1). The details regarding the measurements of each lifestyle were delineated below. Finally, 1634 women were included in this study. Ethical approval was granted by the Human Subjects Research Ethics Committee at the Hong Kong Polytechnic University (Ref.: HSEARS20171229004). All participants provided written informed consent.

### 2.2. Definition and Assessment of the Combined Lifestyle Score

The CLS included eight lifestyle factors: smoking, physical activity, sedentary time, sleep, stress, fatigue, diet, and alcohol. Each component was categorized as either healthy (1 point) or unhealthy (0 point). The CLS was calculated as the total points accumulated across these items, ranging from 0 to 8 points, with higher scores reflecting better health (Table 1).

In the MECH-HK cohort, participants were asked about their smoking habits (current, former, or never) and secondhand smoke exposure (passive exposure to smoking at home or in the workplace). A previous meta-analysis highlighted that active smokers, including both current and former smokers, demonstrated an increased incidence of MetS compared to individuals who never smoked, with a pooled relative risk (RR) and 95% confidence interval (95% CI) of 1.26 (1.10–1.44) [5]. Additionally, findings from a Korean cohort study revealed a higher prevalence of MetS among women exposed to secondhand smoke than individuals who never smoked (5.8% vs. 4.6%; odds ratio (OR) = 1.17; 95% CI = 1.06–1.29; *p* < 0.001) [6]. Therefore, in this study, individuals who had never smoked and those not exposed to secondhand smoke were categorized into the healthy group (1 point).

Additionally, in the MECH-HK cohort, physical activity was evaluated by the Chinese version of the International Physical Activity Questionnaire Short Form (IPAQ-SF) [30]. The reliability and validity of the IPAQ-SF were evaluated in prior investigations. For example, a study including 1270 Hong Kong adults (42.9 ± 14.4 years) found significant associations between reported moderate and vigorous physical activity times in the IPAQ-SF and those measured by an accelerometer (ActiGraph) among women (Spearman correlation *p* < 0.05) [31]. Furthermore, when classifying physical activity according to the World Health Organization (WHO) guidelines (150 min/week of moderate-to-vigorous physical activity), the agreement percentage between the IPAQ-SF and ActiGraph in women was 79% [31]. Another study with 224 Chinese participants in Guangzhou (51–82 years) observed that the intra-class correlation coefficients were 0.81 for moderate physical activity and 0.83 for vigorous physical activity [32].

In this study, physical activity and sedentary time were regarded as two separate components, aligning with a previous study [33]. The WHO guidelines advocate individuals to engage in moderate physical activity ≥150 min/week or vigorous physical activity ≥75 min/week [34]. Thus, in this study, individuals were classified into the healthy physical activity group (1 point) if they satisfied the WHO guidelines for physical activity. Meanwhile, a meta-analysis showed that compared to women in the lowest category of sedentary time (median: 1.50 h/day), women in the intermediate category (median: 4.00 h/day) and highest category (median: 5.75 h/day) had a higher MetS risk, with pooled ORs (95% CIs) of 1.62 (1.21–2.17) (*p* = 0.001) and 2.10 (1.06–4.18) (*p* = 0.030), respectively [35]. Therefore, subjects were categorized into the healthy sedentary time group if they maintained a sedentary time of <4 h/day.

Furthermore, sleep condition was assessed by the Chinese version of the Pittsburgh Sleep Quality Index (PSQI), which has a score of 0–21, with higher scores indicating poorer sleep quality [36]. A PSQI score of >5 was considered as poor sleep quality in prior research [36]; therefore, a PSQI score of ≤5 was defined as healthy in this study. Stress data were collected using the Chinese version of the 14-item Perceived Stress Scale (PSS-14) [37]. The frequency of each item was scored on a 5-point scale, ranging from “never” (scored as 0) to “very often” (scored as 4) [37]. The overall score was then summed up and ranged from 0 to 56, with higher scores indicating a higher stress perception [37]. In this study, consistent with a Chinese study, a PSS-14 score of <25 was defined as healthy [38]. Furthermore, an 11-degree self-perceived fatigue scale ranging from 0 (no fatigue) to 10 (the most severe fatigue) was employed to obtain fatigue data. In this study, a fatigue score less than the median value was classified as healthy.

For diet, according to the recommendations of the Centre for Health Protection of the Department of Health of Hong Kong [39], trained research assistants asked participants about how often they consumed 11 food categories in the past month. These categories included fruits, vegetables, soy-based products, dairy products, cakes, fish, seafood, eggs, red meats, processed meats, and pickled vegetables. The intake frequencies included “never”, “<1 time/month”, “1–3 times/month”, “1–3 times/week”, “4–6 times/week”, and “every day”. The Cronbach’s alpha for the 11-item dietary questionnaire was 0.66, slightly below the commonly utilized threshold of 0.70, though still considered acceptable [40].

In this study, we devised a healthy diet index using most of these food categories. The evidence was based on previous studies focused on the associations between dietary habits and MetS. For example, in previous meta-analyses, the pooled RRs (95% CIs) of MetS for the highest vs. lowest intake categories were 0.81 (0.75–0.88) for fruits [9], 0.89 (0.85–0.93) for vegetables [9], 0.80 (0.72–0.88) for dairy products [11], 0.88 (0.81–0.96) for fish [12], 0.92 (0.88–0.96) for eggs [13], 1.20 (1.06–1.35) for red meats [12], and 1.35 (1.18–1.54) for processed meats [15]. Furthermore, a Korean cohort study indicated that compared to women in the lowest quintile for intake of soy protein, those in the highest quintile had a reduced MetS incidence [10]. Meanwhile, cake was reported to be positively associated with MetS in some studies [16,17]. Moreover, a MetS-related Chinese cohort study suggested that compared to no consumption of pickled vegetables, the risk of diabetes was decreased by consumption of 0–0.5 kg/month (OR = 0.77; 95% CI = 0.63–0.94) and >0.5 kg/month (OR = 0.37; 95% CI = 0.23–0.60) [14]. However, previous studies did not provide robust evidence for the relationships between seafood other than fish and MetS [41] or T2D [42].

As a result, we included a total of ten food categories in the present healthy diet index, which were fruits, vegetables, soy-based products, dairy products, cakes, fish, eggs, red meats, processed meats, and pickled vegetables. Similar to a Chinese study [43], we assigned scores of 1–6 for healthy food (fruits, vegetables, soy-based products, dairy products, fish, eggs, and pickled vegetables), with higher values reflecting more frequent intake. Conversely, for unhealthy food (cakes, red meats, and processed meats), we assigned scores of 1–6, where higher values indicated less frequent intake. The healthy diet index was calculated as the sum of these scores, leading to a range from 10 to 60. A higher score denoted a healthier dietary pattern. In the CLS, a healthy diet was defined as the top tertile of the healthy diet index.

Additionally, the alcohol data were obtained through a question that inquired “How frequently did you consume a can of beer/a glass of wine/a serving of spirits?”. Response options included “never”, “<1 time/month”, “1–3 times/month”, “1–3 times/week”, “4–6 times/week”, and “every day”. The amount of alcohol intake was then computed in grams per day. According to a meta-analysis, women who consumed alcohol < 20 g/day had a reduced MetS risk compared to non-drinkers [18]. Therefore, an alcohol intake of >0 and <20 g/day was defined as healthy in this study [18].

### 2.3. Diagnosis of Metabolic Syndrome

Based on the criteria for clinical diagnosis of MetS proposed by the joint interim statement, MetS was diagnosed as the presence of at least three of the following five conditions [44]: (1) central obesity: waist circumference ≥80 cm; (2) elevated triglycerides: ≥150 mg/dL (1.70 mmol/L) or undergoing treatments; (3) reduced HDL cholesterol: <50 mg/dL (1.29 mmol/L) or receiving treatments; (4) elevated blood pressure: systolic blood pressure ≥130 mmHg, diastolic blood pressure ≥85 mmHg, or hypertension history with treatments; (5) elevated fasting glucose: ≥100 mg/dL (5.60 mmol/L) or drug treatment of elevated glucose.

The measurement of waist circumference was taken at the midpoint between the lower rib margin and the iliac crest using a non-stretchable tape. Additionally, a trained nurse collected finger blood samples. The samples were immediately analyzed using the CardioChek PA device for HDL cholesterol and triglycerides and the ACCU-CHEK Performa device for blood glucose. Participants were instructed to fast for at least 8 h before measurements. Furthermore, blood pressure was measured using an electronic sphygmomanometer after participants had been seated for a minimum of 15 min. Two successive measurements were recorded, and the average of these values was computed and utilized.

### 2.4. Assessment of Covariates

A questionnaire was used to obtain the data on socioeconomic status (age, marital status, education, living condition, and family income), women’s health (menstrual age and menopausal status), and medical history. Height was evaluated using a stadiometer with subjects standing without footwear. Weight was measured utilizing the Inbody 270 body composition measurement machine with participants removing heavy clothing or accessories. Body mass index (BMI) was calculated as weight (kg)/height^2^ (m^2^). General obesity was defined as a BMI ≥25 kg/m^2^ [45].

### 2.5. Statistical Analysis

Baseline characteristics were presented as mean ± SD for continuous variables and number (percentage) for categorical variables. All continuous variables followed the normal distribution assessed by the Q-Q plot. Based on the sample size of each point category, the CLS was categorized into four groups (0–3, 4, 5, and 6–8 points). The ANOVA and Chi-square tests were employed to assess the differences in these baseline characteristics across the four CLS groups for continuous and categorical variables as appropriate.

Logistic regression analyses were conducted to calculate the ORs and 95% CIs for the association between CLS and MetS. Two models were used in multivariable analysis: Model I adjusted for age; Model II adjusted for age, marital status, living condition, educational level, family income, employment status, menstrual age, menolipsis, hypertension, hyperlipidemia, cancer, and general obesity. The lowest CLS group was regarded as the reference group. Furthermore, these analyses were conducted for each CLS component individually, wherein the other components apart from the original covariates were further adjusted in multivariable analyses.

In addition, two sensitivity analyses were performed. First, leave-one-out analyses were carried out by excluding each component one at a time. Second, a weighted CLS was devised utilizing the coefficient (β) and SD of each component in the most-adjusted model. Specifically, the standardized β of each component was first calculated by the formula: standardized β = (β × SD_exposure_)/SD_outcome_. In logistic regression, the SD_outcome_ is π/√3 = 1.8138 [46]. Then, the weight of each component was computed using the formula: Weight*_i_ =* [Standardized β*_i_/*(ΣStandardized β*_i_*)] × 8 (Table S1). The weighted CLS was classified into four groups based on quartiles. Analyses were repeated using the weighted CLS.

All statistical analyses were carried out using SPSS 24.0 (SPSS, Inc., New York, NY, USA). Statistical significance was determined based on a two-sided *p* < 0.05.

## 3. Results

### 3.1. Baseline Characteristics

The baseline characteristics of the 1634 women are shown in Table 2. The average baseline age of total women was 55.9 ± 8.6 years. In comparison with the lowest CLS group (0–3 points), the highest CLS group (6–8 points) had an older average age, a lower BMI value, a higher percentage of women with a low family income and menopausal women, and a decreased proportion of women at an employed status and those with obesity (all *p* < 0.05). In contrast, there was no difference in marital status, living condition, educational level, menstrual age, hypertension, hyperlipidemia, and cancer among the CLS groups (all *p* > 0.05).

For the CLS components, compared to the lowest group (0–3 points), the highest group (6–8 points) had a lower percentage of current/former smokers and secondhand smokers, a higher level of moderate and vigorous physical activity, a lower value of sedentary time, PSQI, and PSS-14, an increased healthy diet index, and a higher percentage of women reporting no or low fatigue and those who were light alcohol drinkers (all *p* < 0.05).

For the components of MetS, compared to the lowest group (0–3 points), the highest group (6–8 points) had a decreased proportion of women with central obesity, a lower level of waist circumference and triglycerides, and a higher level of HDL cholesterol (all *p* < 0.05). However, the levels of SBP, DBP, and fasting glucose were not significantly different across the CLS groups (all *p* > 0.05).

### 3.2. Associations of Combined Lifestyle Score and Its Components with Metabolic Syndrome Incidence

**Figure 2:**
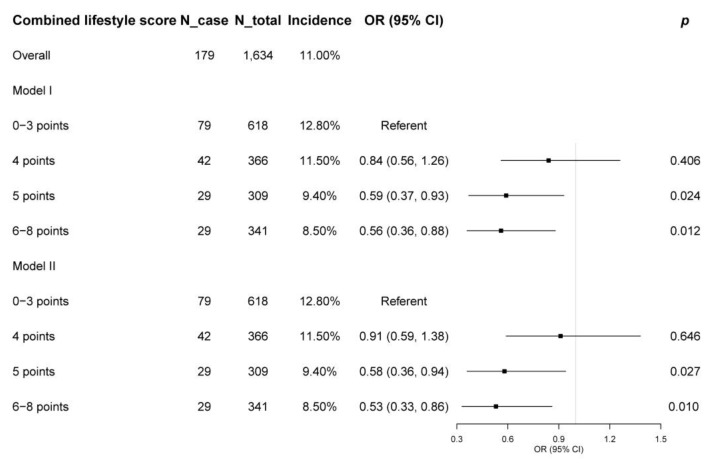
Associations between combined lifestyle score and the incidence of metabolic syndrome. OR, odds ratio; CI, confidence interval. Model I adjusted for age. Model II adjusted for age, marital status, living condition, educational level, family income, employment status, menstrual age, menolipsis, hypertension, hyperlipidemia, cancer, and general obesity.

A total of 179 women (11.0%) developed MetS during an average follow-up time of 1.16 ± 0.18 years. The MetS incidence of the 0–3-point, 4-point, 5-point, and 6–8-point CLS groups were 12.8% (79/618), 11.5% (42/366), 9.4% (29/309), and 8.5% (29/341), respectively (Table 3, Figure 2). Compared to the lowest CLS group (0–3 points), the highest group (6–8 points) had a reduced MetS incidence in both Model I (OR = 0.56; 95% CI = 0.36–0.88; *p* = 0.012) and Model II (OR = 0.53; 95% CI = 0.33–0.86; *p* = 0.010) (Table 3, Figure 2). However, all components of the CLS were not significantly associated with the incidence of MetS (Table 3).

### 3.3. Sensitivity Analysis

In leave-one-out analyses, CLS was always inversely related to MetS after excluding each component one by one (Table S2). In addition, the weight for each component of the CLS is shown in Table S1. Among the eight lifestyle factors, sedentary time, fatigue, and smoking were the top three contributors to the weighted CLS, with weights of 2.452 (30.6%), 2.079 (26.0%), and 1.462 (18.3%), respectively (Table S1). Compared to the lowest weighted CLS group, the highest group had a decreased MetS incidence in Model I (OR = 0.46; 95% CI = 0.29–0.73; *p* = 0.001) and Model II (OR = 0.50; 95% CI = 0.31–0.82; *p* = 0.005) (Table S2).

## 4. Discussion

### 4.1. Summary of Findings

In this study, it was observed that among Hong Kong Chinese women, an eight-item CLS was inversely associated with MetS incidence. The relationship was robust in leave-one-out analyses and when employing a weighted CLS. However, no significant association was observed for all CLS components. These findings implied the potential existence of a synergistic interaction among lifestyles. Individuals who had a high adherence to a healthy lifestyle pattern, characterized by no current/former and secondhand smoking, moderate or vigorous physical activity, low sedentary time, good sleep, low or no stress, low or no fatigue, a healthy dietary pattern, and a light alcohol intake, might have a reduced MetS incidence. The results underscored the significance of adopting a comprehensive lifestyle pattern rather than the individual lifestyle factors in preventing MetS.

### 4.2. Comparisons with Previous Studies and Explanations

Our findings were consistent with previous studies exploring the associations between combined lifestyles and MetS incidence. A 6-year prospective cohort study involving 10,807 Spanish participants found that a nine-item healthy lifestyle score (smoking, physical activity, the Mediterranean diet, alcohol, television exposure, binge drinking, nap, social contact, and working time) was inversely associated with MetS incidence (OR = 0.66; 95% CI = 0.47–0.93; 7–9 vs. 0–3 points) [28]. Likewise, another 6-year prospective cohort study showed that among 3480 Iranian adults, a four-item lifestyle index (smoking, physical activity, obesity, and diet) had an inverse association with MetS incidence [29]. Additionally, a 5-year prospective cohort study with 363 Spanish coronary heart disease patients demonstrated that a Mediterranean lifestyle comprising 27 components was inversely associated with MetS incidence (OR = 0.37; 95% CI = 0.19–0.75; the highest vs. lowest groups) [47]. Nonetheless, these results might not be applied to Chinese populations directly due to the diverse lifestyles and disease characteristics among different ethnic groups [21,22,23]. To our knowledge, there was no cohort study conducted with Chinese people. The only related one is a Chinese cross-sectional investigation involving 532 Chinese adults, which observed an inverse relationship between a five-item healthy lifestyle index (physical activity, diet, smoking, alcohol, and BMI) and MetS prevalence in both men (OR = 0.84; 95% CI = 0.76–0.93) and women (OR = 0.75; 95% CI = 0.65–0.88) [43].

The lifestyle factors included in previous studies were different. Most research incorporated smoking, physical activity, diet, and BMI. Some studies also included sleep, naps, social contact, and working time. In this investigation, BMI was excluded from the CLS due to its significant association with waist circumference, a key parameter used in the diagnosis of MetS [2,44]. In fact, some diagnostic criteria of MetS even incorporated BMI as an assessment parameter [2]. The exclusion of BMI may prevent potential overemphasis on its effect, which could obscure the relationships between other lifestyle factors and MetS. Alternatively, BMI was included as a covariate in multivariable analyses.

In this investigation, to assess the individual contribution of each CLS component on MetS, we calculated a weighted CLS based on the multivariable results of each component. The findings suggested that the weighted CLS might demonstrate a better performance in preventing MetS compared to the original CLS, as evidenced by the lower ORs for the former. However, due to the overlapping 95% CIs between the weighted CLS and the original CLS, assertions regarding statistically significant differences in their effects cannot be made. Additionally, given the complexity involved in calculating the weighted CLS, the use of a CLS with equal weights might be more practical for general application.

Of note, our study observed a high level of physical activity, with over 75% of participants meeting the WHO guidelines of engaging in ≥150 min/week of moderate physical activity or ≥75 min/week of vigorous physical activity [34]. One potential explanation is that the IPAQ-SF itself tends to overestimate physical activity levels [31]. This is exemplified in a Hong Kong study where the IPAQ-SF reported higher levels of both moderate (153.4 ± 178.0 vs. 42.1 ± 23.9 min/day; *p* < 0.001) and vigorous physical activity (9.6 ± 40.7 vs. 1.0 ± 2.8 min/day; *p* < 0.001) compared to ActiGraph measurements [31]. Another potential reason might be the “healthy volunteer effect”. Individuals volunteering for research tend to exhibit healthier lifestyles, potentially biasing our findings. This self-selection bias emphasized the need for caution in interpreting physical activity levels. A third possible reason might be related to the favorable exercise habits among Hong Kong residents. The most updated Hong Kong Thematic Household Survey Report indicated that women aged ≥15 years allocated an average of 0.5 h/day to sports-related activities, amounting to an estimated 210 min/week [48].

Interestingly, our study revealed some counterintuitive findings, for example, the inverse associations of CLS with family income and an employed status. Although these results did not impact our results concerning the CLS-MetS association, they warrant careful discussion. We posit that these findings may stem from intricate interactions involving lifestyle patterns, age, employment status, and family income. Notably, age demonstrated a positive correlation with CLS in our study, a reasonable association given that older individuals often exhibit heightened health awareness, fostering healthier lifestyle patterns compared to their younger counterparts. Furthermore, considering that a substantial proportion of our participants comprised middle-aged and older women (mean age: 55.9 ± 8.6 years), many of them were retired or unemployed. Such individuals might also manifest healthier lifestyle patterns, such as greater engagement in exercise, lower stress and fatigue levels, improved sleep, healthier dietary habits, and a reduced likelihood of excessive alcohol intake than their actively employed counterparts. Moreover, it is logical to assert that retired/unemployed older individuals may have a lower family income compared to their younger counterparts.

### 4.3. Mechanism

The significant correlations between combined lifestyles and MetS can be partially explained by their impacts on some shared factors, such as oxidative stress, inflammatory cytokines, insulin resistance, impaired glucose tolerance, sympathetic nervous system activation, and hormone disturbance. These effects ultimately contributed to the manifestation of MetS components, including increased visceral fat accumulation, dyslipidemia, elevated blood pressure, and heightened blood glucose levels. Of course, certain lifestyles also have their own mechanisms to prevent MetS. On the other hand, these lifestyle factors might also interact with each other, collectively contributing to the development of MetS.

Specifically, the impact of smoking on glucose and lipid metabolism might be attributed, in part, to the stimulation of the sympathetic nervous system and the elevation of circulating levels of insulin-antagonistic hormones, including cortisol and growth hormone [5]. The elevated concentrations of cortisol and exacerbated insulin resistance can also lead to the accumulation of visceral fat mass and an increase in waist circumference [5]. Apart from sympathetic nervous system activation and hormonal disturbance, multiple lines of evidence indicated that smokers experience more pronounced endothelial dysfunction and a concomitant reduction in arterial compliance [5]. This heightened impairment might also result in more severe insulin resistance and compensatory hyperinsulinemia among smokers, potentially leading to elevated levels of blood glucose and blood pressure [5]. Additionally, it was reported that smoking tended to co-occur with a cluster of other unhealthy lifestyles, such as insufficient physical activity, excessive alcohol consumption, and an unhealthy diet [49].

Furthermore, physical activity has been shown to enhance fat metabolism in adipose tissues, particularly in abdominal areas, since the abdominal fat and mesenchymal stem cells derived from fat exhibit heightened sensitivity to physical activity [50]. In addition, physical activity was shown to have the potential to elevate insulin sensitivity, amplify insulin signaling, facilitate glucose transport, and subsequently reduce blood glucose levels [50,51]. Physical activity can also mitigate oxidative stress and inflammation by diminishing the concentrations of some inflammatory markers such as C-reactive protein (CRP), interleukin-6 (IL-6), and IL-18, thereby preventing elevated blood pressure [51]. Other mechanisms pertinent to the preventive role of physical activity in high blood pressure involved the reduction in sympathetic nervous system activity, modulation of the renin–angiotensin–aldosterone system, and attenuation of systemic vascular resistance [51,52]. Moreover, physical activity might contribute to a favorable lipid profile through elevated expression of lipoprotein lipase (LPL) mRNA, increased LPL mass, augmented total LPL activity, and enhanced heparin-releasable LPL activity within skeletal muscle following sustained physical activity [53]. In addition, physical activity has also been documented to have the capacity to diminish BMI, waist circumference, and psychosocial stress [51].

Regarding sleep, inadequate sleep duration might lower leptin and increase ghrelin, leading to increased appetite, weight gain, augmented waist circumference, and disruptions in glycemic control [8]. Moreover, both short and long sleep durations were related to elevated cortisol, IL-6, and tumor necrosis factor-alpha (TNF-a), which could also partially explain insulin resistance, a central characteristic of MetS [8]. Sleep deprivation can also activate the sympathetic system, consequently stimulating the renin–angiotensin–aldosterone system and increasing the synthesis of central catecholamines [54]. This cascade of events results in blood vessel constriction, leading to elevated blood pressure [54]. Earlier investigations have also indicated an association between extended sleep durations and elevated concentrations of total cholesterol, and an increased total/HDL cholesterol ratio, which might be linked to the occurrence of highly fragmented sleep [55]. Additionally, individuals with both insufficient and excessive sleep are more likely to engage in MetS-risk behaviors, such as smoking, physical inactivity, excessive alcohol intake, and low intake of fruits and vegetables [8,54,56].

In addition, stress and fatigue could stimulate the release of catecholamines and cortisol, subsequently increasing blood glucose and inducing insulin resistance [19,57]. Stress and fatigue have also been associated with elevated levels of monocyte chemoattractant protein and inflammatory cytokines, including IL-1 beta and IL-6, thereby causing high blood pressure and dyslipidemia [19,57]. Furthermore, stress was closely associated with the adoption of overeating habits, thereby resulting in elevated body weight and increased waist circumference [19,57].

Regarding dietary factors, some nutrients in fruits and vegetables, such as vitamin C, vitamin E, carotenoids, and flavonoids, could mitigate lipid peroxidation, promote blood vessel relaxation, and improve endothelial function by exerting antiatherogenic, antithrombotic, and anti-inflammatory effects [58,59,60,61]. Furthermore, soy protein could reduce cholesterol, triglyceride, and fat levels by enhancing bile acid excretion and inhibiting hepatic fatty acid synthesis [10]. Meanwhile, the elevated arginine-to-lysine ratio in soy protein, compared to animal sources, suppressed insulin and glucagon secretion, consequently inhibiting lipogenesis [10]. In addition, isoflavones in soy, structurally akin to estrogens, might interact with estrogen receptors and then decrease blood cholesterol [10]. Genistein, a major isoflavone, served as a potent inhibitor of glucose transporter 4-mediated glucose uptake and might stimulate nitric oxide production, exhibiting vasodilatory and antihypertensive effects [10]. Additionally, soy protein induced hormonal and molecular changes associated with adiposity, influencing the expression of sterol regulatory element-binding proteins, pivotal transcription factors governing genes involved in fatty acid and cholesterol synthesis [10].

The protective effects of dairy products stem from the presence of minerals (calcium and potassium), proteins, and fatty acids [11]. Calcium and potassium could impede fat absorption through the formation of insoluble soaps, thereby preventing fat accumulation [11]. Calcium could also bind with bile acids, hindering their absorption and reducing low-density lipoprotein cholesterol [11]. Additionally, calcium exerted blood pressure-lowering effects by decreasing 1,25-dihydroxycholecalciferol, which stimulated calcium influx through vitamin D receptors, thereby promoting contraction and peripheral resistance [11]. Potassium could also lower blood pressure by suppressing proinflammatory processes within vascular smooth muscle cells, diminishing platelet aggregation, and lowering renal vascular resistance [11]. Furthermore, dairy products contain high levels of whey proteins, known to inhibit angiotensin-converting enzyme activity, leading to angiotensin II inhibition [11]. Whey protein also contributed to reducing triglycerides, total cholesterol, and low-density lipoprotein cholesterol by up-regulating the expression of the fatty acid synthase gene, thereby promoting adipocyte lipogenesis [11]. Additionally, dairy products were also rich in fatty acids, such as butyric acid, a representative short-chain fatty acid, exhibiting potential in mitigating weight gain and improving insulin sensitivity [11]. Similarly, medium-chain fatty acids like caprylic acid demonstrated beneficial effects on glucose and energy homeostasis, while capric acid exhibited lipid-lowering properties, reducing total cholesterol and triglyceride levels [11].

A possible rationale for the protective properties of fish was the capacity of fish proteins to retard lipid absorption and synthesis and facilitate lipid excretion [41]. Enhanced insulin sensitivity has also been noted in insulin-resistant individuals consuming fish proteins in comparison to other animal proteins [41]. Additionally, the elevated ratio of unsaturated to saturated fatty acids in fish has been proposed to exhibit anti-inflammatory properties by reducing proinflammatory cytokines, and to exert beneficial effects on insulin sensitivity, vascular function, and blood clotting [42,58,59,60,61]. Furthermore, egg white hydrolysate was able to augment insulin sensitivity and mitigate abdominal obesity [13]. The HDL in egg yolk might contribute to the amelioration in dyslipidemia by influencing fatty acid metabolism [13]. Additionally, phospholipids derived from eggs have been associated with improvements in endothelial vasodilatory function, as well as reductions in waist-to-hip ratio and systolic blood pressure [13].

Red meat was characterized by high levels of total fat, saturated fat, and haem iron [15]. The first two constituents may contribute to the development of obesity, hyperinsulinemia, and hyperglycemia [15]. The pro-oxidative properties of iron fostered oxidative stress and inflammation, potentially causing harm to pancreatic beta cells and subsequently hindering glucose metabolism, along with diminishing the synthesis and secretion of pancreatic insulin [15]. The usage of nitrate as a preservative in processed meat has the potential to transform into nitrosamines, observed to be detrimental to pancreatic cells and linked with insulin resistance [15]. Moreover, pickled foods have been associated with enhanced gut health and a reduction in blood pressure through the supplementation of probiotics like Bifidobacteria [14]. Additionally, pickled vegetables contain alphalinolenic acid, 5-hydroxymethylfurfural, and sitosterol, which exhibited properties of antiatherosclerotic, cardiovascular disease-preventing, anti-inflammatory, and lipid-lowering agents [14]. However, the high salt level in pickled vegetables necessitates further investigations [14].

Moreover, the increased activity of lipoprotein lipase induced by alcohol contributed to the synthesis of HDL cholesterol through the breakdown of very low-density lipoprotein in adipose tissue [62]. Additionally, alcohol diminished the activity of hepatic triglyceride lipase, responsible for HDL removal from the circulation [62]. Alcohol also had the potential to increase the production of apolipoprotein A-I in the liver, a component integral to HDL particles in the periphery [62]. Furthermore, insulin resistance or hyperinsulinemia could influence the functions of lipoprotein lipase in adipose tissue, elevating both the quantity and activities of hepatic triglyceride lipase [62]. Such alterations might lead to a negative correlation between insulin levels and HDL cholesterol levels [62]. Therefore, moderate alcohol consumption might raise HDL cholesterol levels by exerting favorable effects on insulin resistance and hyperinsulinemia [62]. Another conceivable mechanism for the protective impact of moderate alcohol intake was the anti-inflammatory effects of alcohol [63]. Nevertheless, since U-shaped associations between alcohol consumption and insulin levels have been demonstrated, heavy alcohol intake has also been related to elevated levels of fasting glucose and triglycerides, as well as hypertension and central obesity [63,64].

### 4.4. Strengths and Limitations

Although this prospective cohort study might be the first one to reveal an inverse correlation between CLS and MetS incidence in Chinese people, several limitations warrant consideration. Firstly, recall bias might exist due to the reliance on questionnaires for collecting lifestyle data. However, we took measures to reduce this bias by conducting face-to-face interviews instead of self-administered questionnaires. Secondly, in this study, the dietary component was evaluated by developing a healthy diet index based on the consumption frequency of ten specific food items. This approach failed to account for actual intake quantities and omitted some important food categories such as whole grains. Moreover, the Cronbach’s alpha for our dietary questionnaire was 0.66, slightly lower than the commonly accepted threshold of 0.70 [40]. Additionally, the evaluation of validity is constrained by financial and manpower limitations. Therefore, further studies employing systematic questionnaires, such as the food frequency questionnaire or 24 h recalls, are warranted.

Thirdly, as previously mentioned, the use of the IPAQ-SF might lead to an overestimation of physical activity levels. Hence, forthcoming studies are encouraged to utilize accelerometers for a more precise measurement of physical activity levels. Fourthly, fatigue data were assessed using a simple 11-degree self-perceived fatigue scale instead of some systematic tools like the fatigue severity scale. Lastly, given that the study was conducted exclusively with Hong Kong Chinese women, the findings should be interpreted and generalized to other populations with caution.

## 5. Conclusions

In conclusion, this prospective cohort study demonstrated an inverse association of an eight-item CLS, characterized by no current/former and secondhand smoking, moderate or vigorous physical activity, low sedentary time, good sleep, low or no stress, low or no fatigue, a healthy dietary pattern, and minimal alcohol intake, with MetS incidence in Hong Kong Chinese women. The finding was consistent in leave-one-out analyses and when employing a weighted CLS. However, no significant association was found for each individual component. These results suggest the potential presence of a synergistic interaction among the CLS components. Nevertheless, further cohort studies incorporating more detailed data on diet, physical activity, and fatigue are needed to confirm our findings.

## Figures and Tables

**Table 1:** The components and criteria of the combined lifestyle score.

Component	Score
Smoking	
Never smoked and individuals who were not exposed to secondhand smoke	1
Current or former smokers, or individuals who were exposed to secondhand smoke	0
Physical activity	
Moderate physical activity ≥ 150 min/week or vigorous physical activity ≥ 75 min/week	1
Moderate physical activity < 150 min/week and vigorous physical activity < 75 min/week	0
Sedentary time	
<4 h/day	1
≥4 h/day	0
Sleep	
Pittsburgh Sleep Quality Index ≤ 5	1
Pittsburgh Sleep Quality Index > 5	0
Stress	
Perceived Stress Scale-14 < 25	1
Perceived Stress Scale-14 ≥ 25	0
Fatigue	
< the median of an 11-degree self-perceived fatigue scale	1
≥ the median of an 11-degree self-perceived fatigue scale	0
Diet	
The top tertile of a healthy diet index	1
The first and second tertile of a healthy diet index	0
Alcohol	
>0 and <20 g/day	1
0 or ≥20 g/day	0

**Table 2:** Baseline characteristics of participants according to the combined lifestyle score.

Baseline Characteristics ^a^	Total Subjects	Combined Lifestyle Score				*p*
		0–3 Points	4 Points	5 Points	6–8 Points	
N	1634	618	366	309	341	
Age, years	55.9 ± 8.6	54.4 ± 9.0	55.4 ± 8.8	57.1 ± 8.0 *	58.3 ± 7.5 *	<0.001
Marital status						0.087
Never married	424 (25.9%)	171 (27.7%)	89 (24.3%)	81 (26.2%)	83 (24.3%)	
Divorce, separation, or widowhood	193 (11.8%)	61 (9.9%)	47 (12.8%)	50 (16.2%)	35 (10.3%)	
Married or cohabiting	1017 (62.2%)	386 (62.5%)	230 (62.8%)	178 (57.6%)	223 (65.4%)	
Living condition						0.083
Living with spouse, parents, or children	324 (19.8%)	119 (19.3%)	64 (17.5%)	77 (24.9%)	64 (18.8%)	
Others	1310 (80.2%)	499 (80.7%)	302 (82.5%)	232 (75.1%)	277 (81.2%)	
Educational level						0.152
Primary school or lower	94 (5.8%)	38 (6.1%)	17 (4.6%)	20 (6.5%)	19 (5.6%)	
Secondary school or pre-college	947 (58.0%)	336 (54.4%)	223 (60.9%)	173 (56.0%)	215 (63.0%)	
College or higher	593 (36.3%)	244 (39.5%)	126 (34.4%)	116 (37.5%)	107 (31.4%)	
Family income, HKD/month						0.001
≤18,000	444 (27.2%)	197 (31.9%)	103 (28.1%)	132 (42.7%) *	114 (33.4%) *	
>18,000 and ≤40,000	608 (37.2%)	272 (44.0%)	155 (42.3%)	97 (31.4%) *	140 (41.1%)	
>40,000	582 (35.6%)	149 (24.1%)	108 (29.5%)	80 (25.9%)	87 (25.5%)	
Employed	817 (50.0%)	343 (55.5%)	201 (54.9%)	118 (38.2%) *	155 (45.5%) *	<0.001
Menstrual age, years	12.9 ± 1.7	12.9 ± 1.7	12.7 ± 1.5	12.9 ± 1.8	12.9 ± 1.6	0.452
Menolipsis	1218 (74.5%)	431 (69.7%)	270 (73.8%) *	250 (80.9%) *	267 (78.3%) *	0.001
Hypertension	117 (7.2%)	49 (7.9%)	26 (7.1%)	20 (6.5%)	22 (6.5%)	0.641
Hyperlipidemia	302 (18.5%)	118 (19.1%)	59 (16.1%)	57 (18.4%)	68 (19.9%)	0.574
Cancer	100 (6.1%)	40 (6.5%)	26 (7.1%)	15 (4.9%)	19 (5.6%)	0.617
Body mass index, kg/m^2^	22.2 ± 3.0	22.3 ± 3.1	22.2 ± 3.1	22.1 ± 2.8	21.9 ± 3.0 *	0.035
General obesity ^b^	243 (14.9%)	108 (17.5%)	57 (15.6%)	44 (14.2%) *	34 (10.0%) *	0.031
Combined lifestyle score component						
Current or former smoker	59 (3.6%)	39 (6.3%)	12 (3.3%) *	5 (1.6%) *	3 (0.9%) *	<0.001
Secondhand smoker	254 (15.5%)	160 (25.9%)	49 (13.4%) *	31 (10.0%) *	14 (4.1%) *	<0.001
Moderate physical activity, min/week	319.9 ± 315.3	254.2 ± 301.1	336.9 ± 317.2 *	362.5 ± 326.0 *	384.2 ± 306.1 *	<0.001
Vigorous physical activity, min/week	93.2 ± 156.5	56.3 ± 122.0	96.8 ± 153.9 *	113.9 ± 172.1 *	140.2 ± 184.0 *	<0.001
Sedentary time, h/day	8.0 ± 3.6	9.1 ± 3.4	8.2 ± 3.4 *	7.4 ± 3.3 *	6.3 ± 3.6 *	<0.001
Pittsburgh Sleep Quality Index	6.2 ± 3.4	8.0 ± 3.2	6.3 ± 3.3 *	4.9 ± 2.9 *	3.9 ± 2.6 *	<0.001
Perceived Stress Scale-14	24.2 ± 5.8	27.4 ± 5.3	24.4 ± 4.9 *	22.0 ± 5.2 *	20.2 ± 4.9 *	<0.001
No or low fatigue ^c^	809 (49.5%)	100 (16.2%)	174 (47.5%) *	221 (71.5%) *	314 (92.1%) *	<0.001
Healthy diet index	40.0 ± 3.7	37.3 ± 3.4	38.5 ± 3.3 *	40.8 ± 3.5 *	42.1 ± 3.6 *	<0.001
Light alcohol intake ^d^	803 (49.1%)	221 (35.8%)	178 (48.6%) *	161 (52.1%) *	243 (71.3%) *	<0.001
Metabolic syndrome criteria						
Central obesity ^e^	643 (39.4%)	272 (44.0%)	137 (37.5%)	113 (36.7%) *	121 (35.5%) *	0.025
Waist circumference, cm	78.3 ± 8.7	79.0 ± 8.5	78.5 ± 9.7	77.7 ± 7.9 *	77.5 ± 8.3 *	0.034
Triglycerides, mg/dL	136.0 ± 74.3	141.0 ± 81.6	134.6 ± 67.3	134.0 ± 68.7	129.8 ± 72.4 *	0.045
High-density lipoprotein cholesterol, mg/dL	67.0 ± 15.2	65.9 ± 15.5	66.8 ± 14.8	67.0 ± 14.6	69.0 ± 15.3 *	0.030
Systolic blood pressure, mmHg	118.2 ± 16.1	120.0 ± 15.9	119.0 ± 17.1	117.8 ± 16.1	117.2 ± 15.6	0.062
Diastolic blood pressure, mmHg	73.0 ± 9.8	73.4 ± 8.7	73.0 ± 10.1	72.9 ± 9.9	72.7 ± 10.0	0.806
Fasting glucose, mg/dL	5.50 ± 0.55	5.55 ± 0.56	5.49 ± 0.46	5.46 ± 0.63	5.43 ± 0.58	0.743

*: *p* < 0.05 compared to the 0–3-point group. ^a^ Continuous and categorical variables are shown as mean ± standard deviation and number (percentage), respectively. ^b^ General obesity is defined as a BMI ≥25 kg/m^2^. ^c^ No or low fatigue is defined as scoring below the median value of an 11-degree self-perceived fatigue scale. ^d^ Light alcohol intake is defined as consuming >0 and <20 g/day. ^e^ Central obesity is defined as a waist circumference ≥80 cm.

**Table 3:** Associations of combined lifestyle score and its components with the incidence of metabolic syndrome.

Exposure	N_case_	N_total_	Incidence	Model I ^a^		Model II ^b^	
				OR (95% CI)	*p*	OR (95% CI)	*p*
Overall	179	1634	11.0%				
Combined lifestyle score							
0–3 points	79	618	12.8%	Referent		Referent	
4 points	42	366	11.5%	0.84 (0.56, 1.26)	0.406	0.91 (0.59, 1.38)	0.646
5 points	29	309	9.4%	0.59 (0.37, 0.93)	0.024	0.58 (0.36, 0.94)	0.027
6–8 points	29	341	8.5%	0.56 (0.36, 0.88)	0.012	0.53 (0.33, 0.86)	0.010
Smoking							
0 point	35	288	12.2%	Referent		Referent	
1 point	144	1346	10.7%	0.81 (0.54, 1.21)	0.298	0.87 (0.57, 1.32)	0.500
Physical activity							
0 point	40	399	10.0%	Referent		Referent	
1 point	139	1235	11.3%	0.94 (0.67, 1.31)	0.713	0.92 (0.65, 1.32)	0.659
Sedentary time							
0 point	147	1328	11.1%	Referent		Referent	
1 point	32	306	10.5%	0.83 (0.55, 1.25)	0.364	0.79 (0.51, 1.24)	0.307
Sleep							
0 point	100	879	11.4%	Referent		Referent	
1 point	79	755	10.5%	0.88 (0.63, 1.25)	0.484	0.97 (0.67, 1.40)	0.872
Stress							
0 point	86	742	11.6%	Referent		Referent	
1 point	93	892	10.4%	0.92 (0.66, 1.29)	0.634	0.90 (0.63, 1.28)	0.564
Fatigue							
0 point	102	825	12.4%	Referent		Referent	
1 point	77	809	9.5%	0.67 (0.47, 0.95)	0.026	0.79 (0.55, 1.15)	0.228
Diet							
0 point	122	1071	11.3%	Referent		Referent	
1 point	57	563	10.1%	0.99 (0.72, 1.37)	0.959	0.99 (0.70, 1.39)	0.950
Alcohol							
0 point	92	831	11.1%	Referent		Referent	
1 point	87	803	10.8%	0.99 (0.72, 1.35)	0.935	0.99 (0.71, 1.38)	0.950

OR, odds ratio; CI, confidence interval. ^a^ Model I adjusted for age. In the multivariable analysis of each individual component, the other components were further adjusted. ^b^ Model II adjusted for age, marital status, living condition, educational level, family income, employment status, menstrual age, menolipsis, hypertension, hyperlipidemia, cancer, and general obesity. In the multivariable analysis of each individual component, the other components were further adjusted.

## Data Availability

The raw data supporting the conclusions of this article will be made available by the authors on reasonable request.

## Supplementary Materials

The following supporting information can be downloaded at: https://www.mdpi.com/article/10.3390/nu16040547/s1, Table S1. The weight for each component of the combined lifestyle score. Table S2. Sensitivity analyses for the associations of combined lifestyle score with the incidence of metabolic syndrome.

## References

[1] Saklayen M.G. (2018). The Global Epidemic of the Metabolic Syndrome. Curr. Hypertens. Rep..

[2] Huang P.L. (2009). A comprehensive definition for metabolic syndrome. Dis. Model. Mech..

[3] Hirode G., Wong R.J. (2020). Trends in the Prevalence of Metabolic Syndrome in the United States, 2011–2016. JAMA.

[4] Yao F., Bo Y., Zhao L., Li Y., Ju L., Fang H., Piao W., Yu D., Lao X. (2021). Prevalence and Influencing Factors of Metabolic Syndrome among Adults in China from 2015 to 2017. Nutrients.

[5] Sun K., Liu J., Ning G. (2012). Active smoking and risk of metabolic syndrome: A meta-analysis of prospective studies. PLoS ONE.

[6] Kim J.H., Kim B.J., Hyun Y.Y., Kang J.H. (2020). Association between Secondhand Smoke Exposure and Metabolic Syndrome in 118,609 Korean Never Smokers Verified by Self-Reported Questionnaire and Urine Cotinine. Endocrinol. Metab..

[7] Zhang D., Liu X., Liu Y., Sun X., Wang B., Ren Y., Zhao Y., Zhou J., Han C., Yin L. (2017). Leisure-time physical activity and incident metabolic syndrome: A systematic review and dose-response meta-analysis of cohort studies. Metabolism.

[8] Xie J., Li Y., Zhang Y., Vgontzas A.N., Basta M., Chen B., Xu C., Tang X. (2021). Sleep duration and metabolic syndrome: An updated systematic review and meta-analysis. Sleep Med. Rev..

[9] Zhang Y., Zhang D.Z. (2018). Associations of vegetable and fruit consumption with metabolic syndrome. A meta-analysis of observational studies. Public Health Nutr..

[10] Woo H.W., Kim M.K., Lee Y.H., Shin D.H., Shin M.H., Choi B.Y. (2019). Habitual consumption of soy protein and isoflavones and risk of metabolic syndrome in adults ≥ 40 years old: A prospective analysis of the Korean Multi-Rural Communities Cohort Study (MRCohort). Eur. J. Nutr..

[11] Jin S., Je Y. (2021). Dairy Consumption and Risk of Metabolic Syndrome: Results from Korean Population and Meta-Analysis. Nutrients.

[12] Hidayat K., Zhu W.Z., Peng S.M., Ren J.J., Lu M.L., Wang H.P., Xu J.Y., Zhou H., Yu L.G., Qin L.Q. (2022). The association between meat consumption and the metabolic syndrome: A cross-sectional study and meta-analysis. Br. J. Nutr..

[13] Ding J., Zhang Y. (2022). Relationship between Egg Consumption and Metabolic Syndrome. A Meta-Analysis of Observational Studies. J. Nutr. Health Aging.

[14] Cai Y., Yang X., Chen S., Tian K., Xu S., Deng R., Chen M., Yang Y., Liu T. (2023). Regular consumption of pickled vegetables and fermented bean curd reduces the risk of diabetes: A prospective cohort study. Front. Public Health.

[15] Kim Y., Je Y. (2018). Meat Consumption and Risk of Metabolic Syndrome: Results from the Korean Population and a Meta-Analysis of Observational Studies. Nutrients.

[16] Wirfält E., Hedblad B., Gullberg B., Mattisson I., Andrén C., Rosander U., Janzon L., Berglund G. (2001). Food patterns and components of the metabolic syndrome in men and women: A cross-sectional study within the Malmö Diet and Cancer cohort. Am. J. Epidemiol..

[17] DiBello J.R., McGarvey S.T., Kraft P., Goldberg R., Campos H., Quested C., Laumoli T.S., Baylin A. (2009). Dietary patterns are associated with metabolic syndrome in adult Samoans. J. Nutr..

[18] Alkerwi A., Boutsen M., Vaillant M., Barre J., Lair M.L., Albert A., Guillaume M., Dramaix M. (2009). Alcohol consumption and the prevalence of metabolic syndrome: A meta-analysis of observational studies. Atherosclerosis.

[19] Kuo W.C., Bratzke L.C., Oakley L.D., Kuo F., Wang H., Brown R.L. (2019). The association between psychological stress and metabolic syndrome: A systematic review and meta-analysis. Obes. Rev..

[20] Maloney E.M., Boneva R.S., Lin J.M., Reeves W.C. (2010). Chronic fatigue syndrome is associated with metabolic syndrome: Results from a case-control study in Georgia. Metabolism.

[21] Barbaresko J., Rienks J., Nothlings U. (2018). Lifestyle Indices and Cardiovascular Disease Risk: A Meta-analysis. Am. J. Prev. Med..

[22] Zhang Y., Pan X.F., Chen J., Xia L., Cao A., Zhang Y., Wang J., Li H., Yang K., Guo K. (2020). Combined lifestyle factors and risk of incident type 2 diabetes and prognosis among individuals with type 2 diabetes: A systematic review and meta-analysis of prospective cohort studies. Diabetologia.

[23] Zhang Y.B., Pan X.F., Chen J., Cao A., Zhang Y.G., Xia L., Wang J., Li H., Liu G., Pan A. (2020). Combined lifestyle factors, incident cancer, and cancer mortality: A systematic review and meta-analysis of prospective cohort studies. Br. J. Cancer.

[24] Deng Y.Y., Liu Y.P., Ling C.W., Li Y.H., Wu Y.Y., Ke Y.B., Chen Y.M. (2020). Higher healthy lifestyle scores are associated with greater bone mineral density in middle-aged and elderly Chinese adults. Arch. Osteoporos..

[25] Deng Y.Y., Zhong Q.W., Zhong H.L., Xiong F., Ke Y.B., Chen Y.M. (2021). Higher Healthy Lifestyle Score is associated with lower presence of non-alcoholic fatty liver disease in middle-aged and older Chinese adults: A community-based cross-sectional study. Public Health Nutr..

[26] Yang Q., Zhang Q., Ngai F.W., Wang S., Zhang D., Gao Y., Hao C., Wang H.H., Nogueira O.C.B.L., Liu M. (2023). The Multimorbidity and Lifestyle Correlates in Chinese Population Residing in Macau: Findings from a Community-Based Needs Assessment Study. Healthcare.

[27] Yang H.L., Mo B.R., Molassiotis A., Wang M., He G.L., Xie Y.J. (2022). Relationship between multimorbidity and composite lifestyle status in Shenzhen, China. J. Multimorb. Comorb..

[28] Garralda-Del-Villar M., Carlos-Chilleron S., Diaz-Gutierrez J., Ruiz-Canela M., Gea A., Martinez-Gonzalez M.A., Bes-Rastrollo M., Ruiz-Estigarribia L., Kales S.N., Fernández-Montero A. (2019). Healthy lifestyle and incidence of metabolic syndrome in the SUN cohort. Nutrients.

[29] Mirmiran P., Farhadnejad H., Teymoori F., Parastouei K., Azizi F. (2022). The higher adherence to healthy lifestyle factors is associated with a decreased risk of metabolic syndrome in Iranian adults. Nutr. Bull..

[30] Craig C.L., Marshall A.L., Sjöström M., Bauman A.E., Booth M.L., Ainsworth B.E., Pratt M., Ekelund U., Yngve A., Sallis J.F. (2003). International physical activity questionnaire: 12-country reliability and validity. Med. Sci. Sports Exerc..

[31] Lee P.H., Yu Y.Y., McDowell I., Leung G.M., Lam T.H., Stewart S.M. (2011). Performance of the international physical activity questionnaire (short form) in subgroups of the Hong Kong chinese population. Int. J. Behav. Nutr. Phys. Act..

[32] Deng H.B., Macfarlane D.J., Thomas G.N., Lao X.Q., Jiang C.Q., Cheng K.K., Lam T.H. (2008). Reliability and validity of the IPAQ-Chinese: The Guangzhou Biobank Cohort study. Med. Sci. Sports Exerc..

[33] Ra J.S., Kim H. (2021). Combined Effects of Unhealthy Lifestyle Behaviors on Metabolic Syndrome among Postmenopausal Women. Healthcare.

[34] Piercy K.L., Troiano R.P., Ballard R.M., Carlson S.A., Fulton J.E., Galuska D.A., George S.M., Olson R.D. (2018). The Physical Activity Guidelines for Americans. JAMA.

[35] Wu J., Zhang H., Yang L., Shao J., Chen D., Cui N., Tang L., Fu Y., Xue E., Lai C. (2022). Sedentary time and the risk of metabolic syndrome: A systematic review and dose-response meta-analysis. Obes. Rev..

[36] Buysse D.J., Reynolds C.F., Monk T.H., Berman S.R., Kupfer D.J. (1989). The Pittsburgh Sleep Quality Index: A new instrument for psychiatric practice and research. Psychiatry Res..

[37] Cohen S., Kamarck T., Mermelstein R. (1983). A global measure of perceived stress. J. Health Soc. Behav..

[38] Leng M., Wei L., Shi X., Cao G., Wei Y., Xu H., Zhang X., Zhang W., Xing S., Wei H. (2021). Mental distress and influencing factors in nurses caring for patients with COVID-19. Nurs. Crit. Care.

[39] Centre for Health Protection of the Department of Health Guideline on Meal Arrangement and Meal Provision. https://www.chp.gov.hk/files/pdf/gls_for_sectors_on_meal_provision_e.pdf..

[40] Tsang S., Royse C.F., Terkawi A.S. (2017). Guidelines for developing, translating, and validating a questionnaire in perioperative and pain medicine. Saudi J. Anaesth..

[41] Tørris C., Molin M., Cvancarova Småstuen M. (2014). Fish consumption and its possible preventive role on the development and prevalence of metabolic syndrome—A systematic review. Diabetol. Metab. Syndr..

[42] Namazi N., Brett N.R., Bellissimo N., Larijani B., Heshmati J., Azadbakht L. (2019). The association between types of seafood intake and the risk of type 2 diabetes: A systematic review and meta-analysis of prospective cohort studies. Health Promot. Perspect..

[43] Ye Y., Zhou Q., Dai W., Peng H., Zhou S., Tian H., Shen L., Han H. (2023). Gender differences in metabolic syndrome and its components in southern china using a healthy lifestyle index: A cross-sectional study. BMC Public Health.

[44] Alberti K.G., Eckel R.H., Grundy S.M., Zimmet P.Z., Cleeman J.I., Donato K.A., Fruchart J.C., James W.P., Loria C.M., Smith S.C. (2009). Harmonizing the metabolic syndrome: A joint interim statement of the International Diabetes Federation Task Force on Epidemiology and Prevention; National Heart, Lung, and Blood Institute; American Heart Association; World Heart Federation; International Atherosclerosis Society; and International Association for the Study of Obesity. Circulation.

[45] WHO/IASO/IOTF The Asia-Pacific Perspective: Redefining Obesity and Its Treatment. Health Communications Australia Pty Limited. https://iris.who.int/bitstream/handle/10665/206936/0957708211_eng.pdf.

[46] Allison P.D. (2012). Logistic Regression Using SAS: Theory and Application.

[47] Romero-Cabrera J.L., Garcia-Rios A., Sotos-Prieto M., Quintana-Navarro G., Alcala-Diaz J.F., Martin-Piedra L., Torres-Peña J.D., Luque R.M., Yubero-Serrano E.M., Delgado-Lista J. (2023). Adherence to a Mediterranean lifestyle improves metabolic status in coronary heart disease patients: A prospective analysis from the CORDIOPREV study. J. Intern. Med..

[48] Census and Statistics Department Hong Kong Special Administrative Region Thematic Household Survey Report No. 56. https://www.statistics.gov.hk/pub/B11302562015XXXXB0100.pdf.

[49] Tsitsimpikou C., Tsarouhas K., Vasilaki F., Papalexis P., Dryllis G., Choursalas A., Spandidos D.A., Tsatsakis A., Charvalos E., Bacopoulou F. (2018). Health risk behaviors among high school and university adolescent students. Exp. Ther. Med..

[50] Krishnamoorthy Y., Rajaa S., Murali S., Sahoo J., Kar S.S. (2022). Association between behavioural risk factors and metabolic syndrome among adult population in India: A systematic review and meta-analysis of observational studies. Nutr. Metab. Cardiovasc. Dis..

[51] Diaz K.M., Shimbo D. (2013). Physical activity and the prevention of hypertension. Curr. Hypertens. Rep..

[52] Hegde S.M., Solomon S.D. (2015). Influence of Physical Activity on Hypertension and Cardiac Structure and Function. Curr. Hypertens. Rep..

[53] Liang Z.D., Zhang M., Wang C.Z., Yuan Y., Liang J.H. (2022). Association between sedentary behavior, physical activity, and cardiovascular disease-related outcomes in adults—A meta-analysis and systematic review. Front. Public Health.

[54] Wang Y., Mei H., Jiang Y.R., Sun W.Q., Song Y.J., Liu S.J., Jiang F. (2015). Relationship between Duration of Sleep and Hypertension in Adults: A Meta-Analysis. J. Clin. Sleep. Med..

[55] van den Berg J.F., Miedema H.M., Tulen J.H., Neven A.K., Hofman A., Witteman J.C., Tiemeier H. (2008). Long sleep duration is associated with serum cholesterol in the elderly: The Rotterdam Study. Psychosom. Med..

[56] Hu J., Zhu X., Yuan D., Ji D., Guo H., Li Y., He Z., Bai H., Zhu Q., Shen C. (2022). Association of sleep duration and sleep quality with the risk of metabolic syndrome in adults: A systematic review and meta-analysis. Endokrynol. Pol..

[57] Tenk J., Matrai P., Hegyi P., Rostas I., Garami A., Szabo I., Hartmann P., Pétervári E., Czopf L., Hussain A. (2018). Perceived stress correlates with visceral obesity and lipid parameters of the metabolic syndrome: A systematic review and meta-analysis. Psychoneuroendocrinology.

[58] Godos J., Zappala G., Bernardini S., Giambini I., Bes-Rastrollo M., Martinez-Gonzalez M. (2017). Adherence to the Mediterranean diet is inversely associated with metabolic syndrome occurrence: A meta-analysis of observational studies. Int. J. Food Sci. Nutr..

[59] Tsitsimpikou C., Tsarouhas K., Kioukia-Fougia N., Skondra C., Fragkiadaki P., Papalexis P., Stamatopoulos P., Kaplanis I., Hayes A.W., Tsatsakis A. (2014). Dietary supplementation with tomato-juice in patients with metabolic syndrome: A suggestion to alleviate detrimental clinical factors. Food Chem. Toxicol..

[60] Bacopoulou F., Landis G.N., Pałasz A., Tsitsika A., Vlachakis D., Tsarouhas K., Tsitsimpikou C., Stefanaki C., Kouretas D., Efthymiou V. (2020). Identifying early abdominal obesity risk in adolescents by telemedicine: A cross-sectional study in Greece. Food Chem. Toxicol..

[61] Rentoukas E., Tsarouhas K., Kaplanis I., Korou E., Nikolaou M., Marathonitis G., Kokkinou S., Haliassos A., Mamalaki A., Kouretas D. (2012). Connection between telomerase activity in PBMC and markers of inflammation and endothelial dysfunction in patients with metabolic syndrome. PLoS ONE.

[62] Kato I., Kiyohara Y., Kubo M., Tanizaki Y., Arima H., Iwamoto H., Shinohara N., Nakayama K., Fujishima M. (2003). Insulin-mediated effects of alcohol intake on serum lipid levels in a general population: The Hisayama Study. J. Clin. Epidemiol..

[63] Koppes L.L., Dekker J.M., Hendriks H.F., Bouter L.M., Heine R.J. (2005). Moderate alcohol consumption lowers the risk of type 2 diabetes: A meta-analysis of prospective observational studies. Diabetes Care.

[64] Sun K., Ren M., Liu D., Wang C., Yang C., Yan L. (2014). Alcohol consumption and risk of metabolic syndrome: A meta-analysis of prospective studies. Clin. Nutr..

