# Supplementary material for: Combined Influence of Eight Lifestyle Factors on Metabolic Syndrome Incidence: A Prospective Cohort Study from the MECH-HK Study
Source: Nutrients. 2024 Feb 16;16(4):547. doi: 10.3390/nu16040547 (PMC10892175; doi:10.3390/nu16040547)
Supplement: Supplementary file 1 [file nutrients-16-00547-s001.zip › nutrients-2835180-supplementary.pdf]

Table S1. The weight for each component of the combined lifestyle score

Component	β [a]	Standard error	Standard β [b]	Proportion [c]	Weight [d]
Smoking	-0.145	0.214	-0.689	18.3%	1.462
Physical activity	-0.079	0.180	-0.319	8.5%	0.677
Sedentary time	-0.230	0.225	-1.156	30.6%	2.452
Sleep	-0.030	0.187	-0.125	3.3%	0.266
Stress	-0.104	0.181	-0.420	11.1%	0.891
Fatigue	-0.230	0.191	-0.980	26.0%	2.079
Diet	-0.011	0.174	-0.042	1.1%	0.090
Alcohol	-0.011	0.169	-0.040	1.1%	0.085

[a]: Age, marital status, living condition, educational level, family income, employment status, menstrual age, menolipsis, hypertension, hyperlipidaemia, cancer, general obesity, and the other components of the combined lifestyle score were adjusted in multivariable analyses.

[b]: Standardized β = β × SD of the exposure/SD of the outcome; In logistic regression, the SD of the outcome is $\pi / \sqrt{3} = 1.8138$.

[c]: Proportion = Standardized $\beta_i / (\sum \text{Standardized } \beta_i)$.

[d]: $\text{Weight}_i = [\text{Standardized } \beta_i / (\sum \text{Standardized } \beta_i)] \times 8$.

Table S2. Sensitivity analyses for the associations of combined lifestyle score with the incidence of metabolic syndrome

Exposure	N_{case}	N_{total}	Incidence	Model I [a]		Model II [b]	
				OR (95% CI)	P	OR (95% CI)	P
Overall	179	1,634	11.0%				
Combined lifestyle score							
0-3 points	79	618	12.8%	Referent		Referent	
4 points	42	366	11.5%	0.84 (0.56, 1.26)	0.406	0.91 (0.59, 1.38)	0.646
5 points	29	309	9.4%	0.59 (0.37, 0.93)	0.024	0.58 (0.36, 0.94)	0.027
6-8 points	29	341	8.5%	0.56 (0.36, 0.88)	0.012	0.53 (0.33, 0.86)	0.010
Combined lifestyle score (no smoking)							
0-2 points	70	552	12.7%	Referent		Referent	
3 points	46	374	12.3%	0.93 (0.62, 1.39)	0.727	0.97 (0.64, 1.49)	0.904
4 points	35	344	10.2%	0.65 (0.42, 1.01)	0.056	0.63 (0.40, 1.01)	0.053
5-7 points	28	364	7.7%	0.53 (0.33, 0.84)	0.007	0.52 (0.32, 0.85)	0.009
Combined lifestyle score (no physical activity)							
0-2 points	61	476	12.8%	Referent		Referent	
3 points	49	400	12.3%	0.95 (0.63, 1.43)	0.807	1.01 (0.66, 1.55)	0.956
4 points	38	388	9.8%	0.65 (0.42, 1.00)	0.053	0.63 (0.40, 1.00)	0.050
5-7 points	31	370	8.4%	0.57 (0.36, 0.90)	0.016	0.58 (0.36, 0.94)	0.027
Combined lifestyle score (no sedentary time)							
0-2 points	48	366	13.1%	Referent		Referent	
3 points	35	307	11.4%	0.84 (0.53, 1.34)	0.469	0.76 (0.46, 1.25)	0.276
4 points	45	380	11.8%	0.83 (0.53, 1.29)	0.405	0.82 (0.52, 1.29)	0.386
5-7 points	51	581	8.8%	0.55 (0.36, 0.84)	0.006	0.52 (0.33, 0.81)	0.004
Combined lifestyle score (no sleep)							
0-2 points	64	414	15.5%	Referent		Referent	
3 points	38	361	10.5%	0.67 (0.44, 1.04)	0.074	0.60 (0.38, 0.95)	0.028
4 points	40	437	9.2%	0.50 (0.33, 0.77)	0.001	0.47 (0.30, 0.73)	0.001
5-7 points	37	422	8.8%	0.44 (0.28, 0.68)	<0.001	0.42 (0.26, 0.67)	<0.001
Combined lifestyle score (no stress)							
0-2 points	57	435	13.1%	Referent		Referent	

Exposure	N_{case}	N_{total}	Incidence	Model I [a]		Model II [b]	
				OR (95% CI)	P	OR (95% CI)	P
3 points	46	385	11.9%	0.95 (0.62, 1.44)	0.802	0.88 (0.56, 1.37)	0.561
4 points	38	393	9.7%	0.63 (0.41, 0.98)	0.040	0.57 (0.36, 0.91)	0.017
5-7 points	38	421	9.0%	0.62 (0.40, 0.96)	0.031	0.63 (0.40, 0.98)	0.048
Combined lifestyle score (no fatigue)							
0-2 points	52	432	12.0%	Referent		Referent	
3 points	43	362	11.9%	1.03 (0.66, 1.58)	0.911	0.91 (0.58, 1.44)	0.686
4 points	46	436	10.6%	0.82 (0.53, 1.25)	0.350	0.74 (0.47, 1.16)	0.187
5-7 points	38	404	9.4%	0.67 (0.43, 0.99)	0.048	0.58 (0.36, 0.93)	0.023
Combined lifestyle score (no diet)							
0-2 points	49	380	12.9%	Referent		Referent	
3 points	44	366	12.0%	0.96 (0.62, 1.49)	0.861	0.86 (0.54, 1.37)	0.530
4 points	45	396	11.4%	0.85 (0.55, 1.31)	0.456	0.77 (0.49, 1.22)	0.266
5-7 points	41	492	8.3%	0.55 (0.35, 0.85)	0.008	0.49 (0.31, 0.79)	0.003
Combined lifestyle score (no alcohol)							
0-2 points	48	368	13.0%	Referent		Referent	
3 points	50	430	11.6%	0.95 (0.62, 1.46)	0.825	0.88 (0.56, 1.39)	0.588
4 points	40	365	11.0%	0.78 (0.50, 1.23)	0.288	0.76 (0.47, 1.22)	0.256
5-7 points	41	471	8.7%	0.56 (0.36, 0.88)	0.011	0.54 (0.34, 0.86)	0.009
Weighted combined lifestyle score							
The first quartile (0-2.314)	55	397	13.9%	Referent		Referent	
The second quartile (2.314-3.815)	50	420	11.9%	0.88 (0.58, 1.33)	0.542	0.94 (0.61, 1.46)	0.778
The third quartile (3.815-5.199)	41	410	10.0%	0.63 (0.41, 0.97)	0.035	0.67 (0.42, 1.06)	0.086
The fourth quartile (5.199-8)	33	407	8.1%	0.46 (0.29, 0.73)	0.001	0.50 (0.31, 0.82)	0.005

OR, odds ratio; CI, confidence interval.

[a]: Model I adjusted for age.

[b]: Model II adjusted for age, marital status, living condition, educational level, family income, employment status, menstrual age, menolipsis, hypertension, hyperlipidaemia, cancer, and general obesity.
